# In Memoriam, William H. Jackson, M. D.

**Published:** 1904-02

**Authors:** Alvin A. Hubbell


### In Memoriam, William H. Jackson, M. D.

BY ALVIN A. HUBBELL, M. D.

Dr. William H. Jackson closed his eyes in death, at 6 p. m., on November 28, 1903, after an illness of eight days from pneumonia,—that dread disease which, in its fatal ravages, so frequently invades the homes of both high and low, and spreads sorrow and desolation in its path. Already, however, had his constitution been made vulnerable to morbific agents by diabetes mellitus, from which he had suffered, and with which he had courageously, and with more or less success contended, for four years. He was 62 years of age, and was at the zenith of his professional career.

Dr. Jackson's life was marked by various activities. He came from a good family, and first saw the light of day on August 26, 1841, in Clarkson, Monroe County, N. Y., where his parents then lived. While quite young his family moved to Sandusky, N. Y., where he spent his boyhood days. Here he received a common school education, and afterwards attended the State Normal School at Albany, N. Y., from which he graduated in due time. He also took a business course in the Eastman Business College at Poughkeepsie, N. Y. Subsequently his time was divided between teaching in our public schools and business pursuits, until 1869, when, through the influence of his brother, B. F. Jackson, state engineer and surveyor of South Carolina, he went to Columbia, S. C.

In 1863, he was married to Miss Mary Hyde of Sandusky, N. Y., and they resided in that place until she died, in 1870, except that he was south for about a year before her death. After her death, he continued his residence in Columbia, S. C., until 1877. During a part of this time, he was engaged in teaching, being employed as principal of the public schools of that city. There, also, for some time, he was the proprietor and editor of a Republican newspaper, the management of which, during the exciting days of reconstruction after the Civil War, required vast amount of courage as well as good judgment. During the early part of this period, he also found time to study medicine, and graduated from the University of South Carolina, in 1873. He did not, however, engage in the practice of medicine while living south.

In 1877, he returned north and renewed his studies in medicine, spending a good share of that year in the Buffalo General Hospital. In the meantime, he married Miss Frances Rockwell, of Pike, N. Y., and in 1878, located at Springville, N. Y., for the practice of his profession. He at once obtained a substantial following. During the quarter of a century in which he resided in that town, he became recognised as one of the most prominent physicians and surgeons of Western New York, and was placed in many positions of trust and honor. For fourteen years he was surgeon to the Buffalo, Rochester & Pittsburg R. R., and for many years was a member of the United States pension examing board, of which he was president at the time of his death. He was an enthusiastic Free Mason, and had been admitted to all its degrees as high as the Knights Templar.

He was a member of numerous medical societies, of which the most important were the Medical Society of the County of Erie, New York State Medical Association, American Medical Association, and the National Association of Railroad Surgeons, in some of which he held honored official positions.

Dr. Jackson was quiet and unassuming in his bearing, and yet he had a strong personality. He led a temperate and exemplary life, and was most genial and companionable as a fellow-practitioner and friend. He was a man of sound judgment, and this was reinforced by a high and cultivated intellect. His conscience was sensitive, and his sympathies were tender and deep. In short, his make-up was such that he strikingly illustrated the best type of the time honored family physician,—ever attentive, ever kind, ever ready to supply all available means of relief, even to the calling to his assistance of colleagues who might possibly be able to contribute thereto, whenever difficulties and doubts arose.

Dr. Jackson showed true professional spirit by his dignified and differential intercourse with medical men, by his close affiliations with medical organizations, by his familiarity with the best and most recent literature, and by his occasional resort to clinical centers to witness the new and improved methods of our art. But while he was eager to profit by the experience and suggestions of his fellow practitioners, he was very reticent about urging himself forward and about recording his own experience and opinions for the benefit of others. This is to be regretted, for he had an abundance that would have been helpful and useful. Yet he was always ready to lend a helping hand, and the young physician found in him a true and efficient friend.

To the country practitioner are presented exigencies, emergencies and temptations which put him to the severest test of skill, intelligence and honor. Dr. Jackson was subjected to these tests and was never found lacking. The country practitioner has opportunities for professional growth and intelligence such as are found in no other field of practice which, if used properly, make him a pillar of strength in our ranks. Dr. Jackson was one of these. The capable, honest, faithful country practitioner has an access to the hearts of the people, and wins from them a confidence, devotion and affection such as nowhere else obtains. Dr. Jackson enjoyed this privilege and reward

Dr. Jackson left a widow and five children: Mrs. S. R. Shuttleworth, of Buffalo; Dr. W. Heman Jackson (dentist), of Galeton, Pa., and Lucien P. Jackson, of Warren, Pa., by his first wife, and Fletcher R., and Lawrence C. Jackson, by his second wife. Besides these, he leaves two brothers and two sisters. In his death the family loses its dearly beloved, and the community a much esteemed and trusted physician and friend. Sincere sympathy is extended to all.

